# Near-infrared intraoperative imaging with indocyanine green is beneficial in video-assisted thoracoscopic segmentectomy for patients with chronic lung diseases: a retrospective single-center propensity-score matched analysis

**DOI:** 10.1186/s13019-020-01310-z

**Published:** 2020-10-07

**Authors:** Zhengcheng Liu, Rusong Yang, Hui Cao

**Affiliations:** 1 Department of Thoracic Surgery, Nanjing Chest Hospital, Treatment and Research Center for Pulmonary Nodule in Nanjing Medical University, Nanjing, 210029 China; 2 Affiliated Nanjing Brain Hospital, Nanjing Medical University, Nanjing, 210029 China

**Keywords:** Near-infrared intraoperative imaging, Intersegmental border, Segmentectomy, Chronic lung diseases

## Abstract

**Objective:**

To investigate whether video-assisted thoracoscopic segmentectomy using near-infrared fluorescence imaging had better intersegmental plane visualization and peri-operative outcome in patients with chronic lung diseases.

**Methods:**

Data were collected retrospectively from March 2014 and August 2019. A total of 92 patients with pulmonary nodules underwent near-infrared fluorescence guided uni-port thoracoscopic segmentectomy(NIF-VATS), 149 patients underwent thoracoscopic segmentectomy with inflation-deflation method(ID-VATS). After 1:1 propensity matching, perioperative outcomes between NIF-VATS and ID-VATS was compared.

**Results:**

Incision size was 3 cm in both group.Mean operative time was 79 min in NIF-VATS group and 96 min in ID-VATS group. The intersegmental plane was not clear in 33 cases of ID-VATS group, and no clear boundary was found after prolonged waiting time. Emphysema or pulmonary bullae could be found in chest CT scan in these patients, they all were diagnosed as chronic obstructive pulmonary disease. In NIF-VATS group, the intersegmental plane was not clear in 8 cases. Under the guidance of three-dimensional reconstruction and preoperative positioning, the oncological margin length of both groups met the requirements of surgical quality control. The intraoperative blood loss, number of lymph node resection, showed no statistical difference between the two groups. Postoperative air leakage was more often observed in ID-VATS group. The postoperative drainage duration, postoperative hospitalization time was shorter in ID-VATS group.

**Conclusions:**

Compared with inflation-deflation method, segmentectomy using NIF imaging is feasible for patients with chronic lung diseases with better intersegmental plane, shorter operation time, less complications, it might lead to faster recovery.

## Introduction

Lung cancer is a malignant tumor with the highest incidence and mortality in the world [1]. With the popularization of low dose computed tomography screening, the epidemiological characteristics of lung cancer have changed greatly [2]. Segmentectomy is recommended for surgical treatment of early stage lung cancer, especially for patients with limited pulmonary function preserved [3]. One of the most important factors for segmentectomy is to identify the accurate intersegmental plane, which is particularly important to ensure sufficient distance between the cutting edges and lesion [4]. Inflation-deflation method is the widely used, however, the intersegmental plane was usually hard to be identified clearly using inflation-deflation method in patients with chronic lung diseases due to decreased lung compliance. Near-Infrared Fluorescence (NIF) imaging with indocyanine green (ICG) is a recent advancement in segmentectomy with high success rates of intersegmental border visualization [5], the brightness of ICG fluorescence mainly depends on both the ICG concentration in blood vessels and the blood flow in the pulmonary artery, it might be beneficial in VATS segmentectomy for patients with chronic lung diseases.

Few studies compared near-infrared fluorescence (NIF) imaging with indocyanine green and inflation-deflation method in identifying intersegmental border. The objective is to investigate whether near-infrared fluorescence imaging with indocyanine green had better intersegmental border visualization and peri-operative outcome in patients with chronic lung diseases underwent segmentectomy.

## Patients and methods

### Patient selection

A retrospective analysis was performed, a total of 92 patients with chronic lung diseases underwent thoracoscopic segemtectomy with near-infrared fluorescence (NIF) imaging. The first near-infrared fluorescence guided thoracoscopic segmentectomy was performed in September 2017, the first thoracoscopic segmentectomy using inflation-deflation method was performed in March 2014, and the initial 30 cases were not included due to the learning curve effect [6], a total of 149 patients with chronic lung diseases were included in control group. All surgeries were performed by highly-experienced experts in this specialized procedure(Highly experienced surgeon was defined as surgeon achieved more than 200 cases of segmentectomy, including sub-segmentectomy and combined segmentectomy.). All surgeries were completed through one single incision. Consultants in our department all agreed that either technique was suitable for each patient. Cases with conversion to thoracotomy or lobectomy were excluded.

This study for the application of thoracoscopic segmentectomy and near-infrared fluorescence imaging was approved by by the institutional review board at Nanjing Chest Hospital(number of the ethics approval: 2014-KL002–02 for uniportal thoracoscopic segmentectomy, 2017-KL-002-03 for near-infrared fluorescence guided thoracoscopic segmentectomy), written informed consent about operative techniques and to the data-use agreement was obtained from all patients before surgery.

The inclusion criteria for NIF-VATS or ID-VATS segmentectomy in patients with chronic lung diseases: Pulmonary nodules with a diameter of less than or equal to 2 cm in thin-slice chest CT and have at least one of the following characteristics: 1. adenocarcinoma in situ, 2 composition of ground glass appearance is greater than or equal to 50%, 3. lesion doubling time is greater than or equal to 400 d; Based on preoperative CT findings, patients with comorbid COPD(emphysema or chronic bronchitis), ILD(idiopathic pulmonary fibrosis (IPF) and interstitial pneumonia(IP), or CPFE(Combined pulmonary fibrosis and emphysema).

**Fig. 1:**
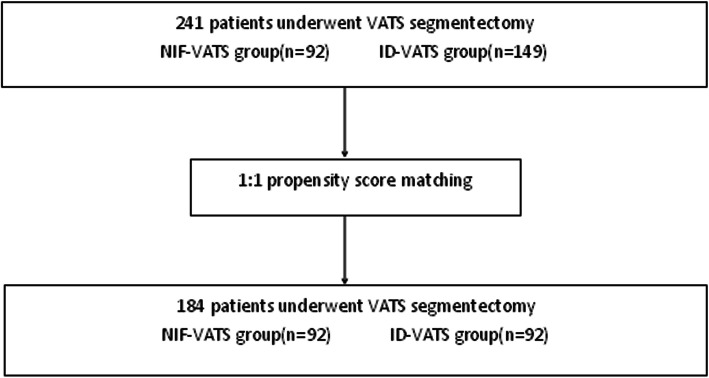
The flowchart of the study

Exclusion criteria were pulmonary nodules that cannot be removed by segmental pulmonary resection, multiple primary tumors require surgery other than pulmonary segmentectomy, wedge resection, patients unable to tolerate surgery(Fig. 1).

Segmentectomy was categorized into simple or complex from the standpoint of technical aspect: resection of superior segment of the lower lobe or the lingular segment was defined as simple. Complex segmentectomy was resection of segment other than that to be resected in the simple segmentectomy, beside, complex segmentectomy was defined as resection of a segment that had more than one intersegmental plane [7]. Two or more intersegmental planes would make segmentectomy more technically diffificult, the operation time would be longer with lower success rate of inter-segmental visualization, and higher rate of post-operative complications.

### Quality control standard

**Fig. 2:**
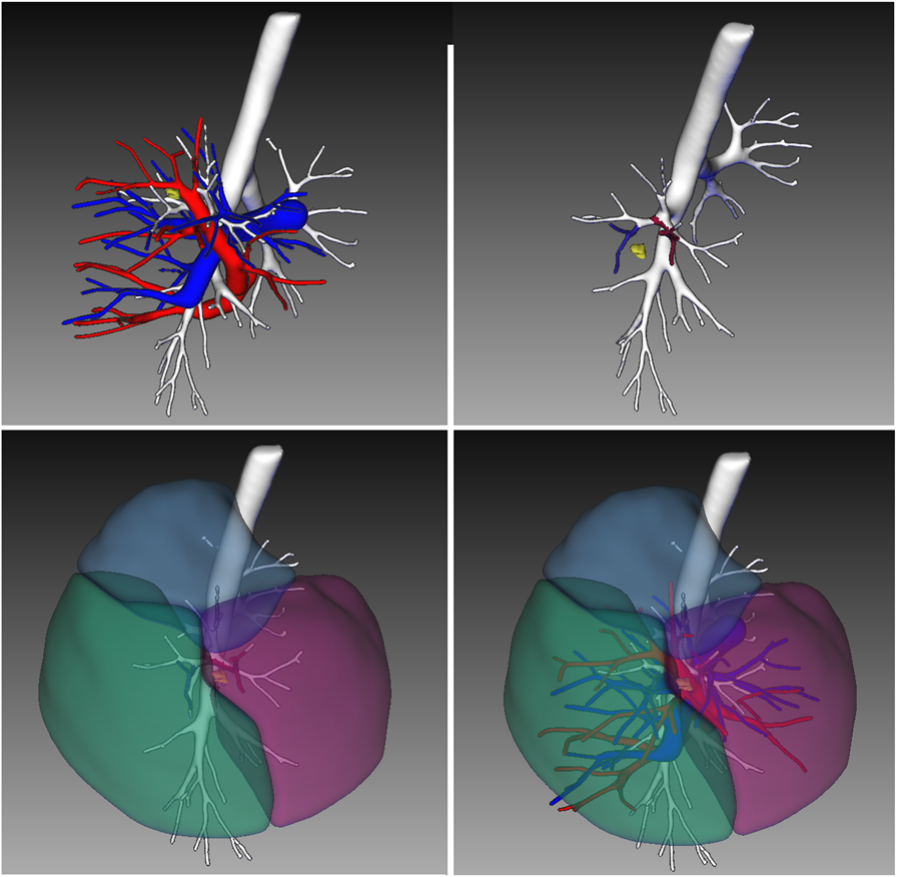
Three-dimensional reconstruction was completed before surgery

All patients underwent contrast-enhanced computer tomography(CT), three-dimensional reconstruction was completed using EDDA-IQQA software or 3D-slicer(before November 2017). CT guided preoperative localization was performed(Fig. 2), except lesion located in apical, lingular or superior segment. Oncological margin length should be larger than 2 cm or maximal diameter of lesion.

### Anaesthesia and incision

The anaesthesia procedures were the same in both groups. All patients received combined intravenous and inhalation anesthesia, with double-lumen endotracheal intubation. The patient was kept in lateral decubitus position, both cranial and caudal side was pushed down like a fold knife to make intercostal space wider, uni-portal VATS was applied. All patients were extubated after operation. Postoperative patient-controlled analgesia was provided.

A single incision, about 3 cm long, was performed at the fifth intercostal space along the anterior axillary line. Plastic wound protector was used. During operation, a 10-mm 30-degree thoracoscope (Pinpoint, Novadaq Technologies ULC, Stryker, for NIF-VATS segmentectomy; Karl Storz for ID-VATS segmentectomy) was placed at the superior side of the incision, several thoracoscopic instruments were simultaneously fitted into the uni-port beneath the thoracoscope.

### Segmentectomy using inflation-deflation method or NIF imaging

Following ligation of the corresponding segmental artery, bronchus, and vein according to preoperative CT and three-dimensional reconstruction, intersegmental plane was identified by inflation-deflation method or near-infrared fluorescence imaging.

Using inflation-deflation method, the collapsed pulmonary tissue was initiated to fully re-expand with controlled airway pressure under 20 cm H_2_O by pure oxygen, followed by single lung ventilation. After an interval of approximately 20 min, an irregularly curved demarcation was identified naturally between the deflated preserving segments and the inflated target segment.

We observed ICG fluorescence using a near-infrared thoracoscope. ICG was reconstituted into distilled water to produce a 2.5 mg/ml solution, and a volume of 6-10 mL (dose of 15 mg to 20 mg) was injected in a peripheral intravenous catheter, followed by 10 ml of saline solution. The intersegmental plane of the targeted segment became clearly visible on infrared thoracoscopy about 15 s after injection, and could be marked using electrocautery.

A monopolar cautery hook was used to divide the pulmonary parenchyma of the intersegmental plane from the central side toward the peripheral side along the marked line, and anatomical segmentectomy was achieved using autosuture’s cartridges.

When the ICG fluorescence was too dim to enable any visualization of the intersegmental border, or only part of the intersegmental border could be found in NIF-VATS group; the target segment was not demarcated from the rest of the lung in ID-VATS group, successfully resection along the border could not be achieved, it was regarded as “unclear intersegmental boarder”. Then intersegmental vein should be fully dissected, the target segment was excised along it.

Surgical margin, the distance between the cut end uncovered by visceral pleura and the tumor margin, was evaluated macroscopically to confirm that the surgical margin was not less than the maximum tumor diameter or 20 mm. If tumor free margin was not achieved, extended segmentectomy would be performed.

Lymph node sampling was performed when intra-operative pathology showed minimal invasive adenocarcinoma(MIA) or invasive adenocarcinoma(IAC).

To control air leakage after segmentectomy, partial closure of the visceral pleura, the use of fibrin glue, or reinforcement with a absorbable pad on the intersegmental plane were allowed.

Extubation criteria included: 1.hemodynamic stability; 2.stable spontaneous ventilation (respiratory rate < 30/min, increase in respiratory rate < 10/min); 3.normal arterial blood gas analysis with low-flow oxygen inhalation (PaO_2_ > 80 mmHg, increase in PaCO_2_ < 10 mmHg, pH > 7.30, SpO_2_ > 90%); 4.clear consciousness.

### Chest tube remove

One 22F chest drain was inserted to thoracic cavity at the end of the operation, it was placed at posterior part of the uniportal incision. Drain removal criteria were as follows: no observed air leak and total drainage less than 200 ml in 24 h; normal chest roentgenograph; normal vital signs; good overall medical status. No patient was discharged with a chest tube in situ.

### Postoperative treatment and follow-up

Patients with ASA score > 2, or age > 70 years would go to ICU after operation. Mean stay in ICU was 2 days. Respiratory rehabilitation included: oxygen administration, in patients who have an elevated PaCO_2_ preoperatively, oxygen saturation is maintained at 90% or less, preserving the hypoxic drive to breathe; early physical exercise; pain control; prevention of cardiac arrhythmias, myocardial infarction, deep venous thrombosis and pulmonary thoromboembolism.

Chest radiography was performed at the first day postoperatively and every 3 days until discharge. Drinking and meal intake were resumed after bowel sounds returned with no nausea or vomiting in both groups.

Postoperative wound pain was monitored using the Visual Analogue Scales(VAS). The scale is an integer scale of 0–10, where 0 is no pain and 10 is the worst pain imaginable. VAS was evaluated on postoperative days 1, 3, 7, 14, 30, 60.

### Statistical analysis

SPSS 16.0 for Windows (IBM, Armonk, NY) was used for analysis. To minimize the impact of potential confounders and selection bias, propensity score analysis was used to compensate for the differences in baseline patient characteristics between the two groups of patients. Patients in the two groups were 1:1 matched using the nearest propensity score on the logit scale. Variables that could influence the outcomes of treatment were matched, including age, gender, body mass index (BMI), ASA status class, and maximal lesion size. After PSM, differences in continuous and categoric clinical characteristics were compared.

Continuous data are presented as mean and SD and were analyzed with two-sample Student’s t tests for independent data. Categorical variables are given as a count and percentage of patients and analyzed with the χ^2^ or Fisher’s exact test. All tests were two-sided, *P*-values < 0.05 were considered statistically significant. SPSS Statistics 16.0 (IBM Corp, Armonk, NY) was used for statistical evaluations.

## Results

Between September 2017 and August 2019, a total of 122 patients with chronic lung diseases underwent uni-portal thoracoscopic segemtectomy with near-infrared fluorescence (NIF) imaging, 30 cases were not included due to the learning curve effect. A total of 149 patients with chronic lung diseases underwent uni-portal thoracoscopic segemtectomy using inflation-deflation method was included in control group. Before matching, there were no significant differences between the two groups in terms of gender, age, smoking history, pulmonary function(FEV1, %FEV1, FEV1/FVC, %DLCO),preoperative CT findings, BMI, ASA score, comorbidity, maximal tumor size on CT scan, the lung lobe where lesion located, type of segmentectomy. After 1:1 matching, 184 patients remained for analysis, baseline demographic and clinical variables were comparable between the two groups (Table 1). Position of lesion and extent of surgical area included RS^1^, RS^2^, RS^3^, RS^2^b + S^3^a, RS^1^a + S^2^a, RS^3^a + S^1^b, RS^6^, RS^7 + 8^, RS^9 + 10^, RS^7–10^, LS^1 + 2 + 3^, LS^1 + 2^, LS^1 + 2^b + c, LS^4^ + S^1 + 2^c, LS^3^, LS^4 + 5^, LS^6^, LS^8^, LS^9 + 10^, LS^7–10^ .

**Table 1:** Characteristics of patients who underwent uni-port video-assisted thoracoscopic segmentectomy using near-infrared fluorescence imaging or inflation-deflation method(FEV1: percentile forced expiratory volume in 1 s; %FEV1: percentile forced expiratory volume in 1 s; FVC: forced vital capacity; %DLCO: percentile diffusing capacity of carbon monoxide)

	Before propensity score matching			After propensity score matching		
	NIF-VATS(*n* = 92)	ID-VATS(*n* = 149)	*P*-value	NIF-VATS(n = 92)	ID-VATS(n = 92)	*P*-value
Gender			0.54			0.66
Male	43(46.7%)	72(48.3%)		43(46.7%)	45(48.9%)	
Female	49(53.3%)	77(51.7%)		49(53.3%)	47(51.1%)	
Age	69.8 ± 5.2	71.3 ± 6.1	0.36	69.8 ± 5.2	70.9 ± 3.5	0.79
Smoking History (no. of smokers) (%)	47(51.1%)	73(49.0%)	0.43	47(51.1%)	46(50.0%)	0.55
FEV1(L)	1.69 ± 0.54	1.53 ± 0.67	0.42	1.69 ± 0.54	1.58 ± 0.41	0.73
%FEV1 (%)	82.1 ± 17.8	83.5 ± 18.6	0.72	82.1 ± 17.8	82.7 ± 16.9	0.81
FEV1/FVC (%)	63.8 ± 11.1	68.4 ± 10.1	0.44	63.8 ± 11.1	65.5 ± 9.2	0.64
%DLCO (%)	77.1 ± 18.3	78.6 ± 17.0	0.79	77.1 ± 18.3	77.9 ± 16.3	0.83
Body mass index (kg/m^2^) (median and range)	22.8 ± 2.7	23.6 ± 3.3	0.31	22.8 ± 2.7	23.2 ± 2.1	0.58
Comorbid Chronic lung disease			0.83			0.95
COPD	90(97.8)	147(98.6%)		90(97.8)	90(97.8)	
IP	3(3.2%)	5(3.3%)		3(3.2%)	2(2.2%)	
CFPE	1(1.0%)	2(1.3%)		1(1.0%)	1(1.0%)	
IPF	1(1.0%)	3(1.5%)		1(1.0%)	1(1.0%)	
Other Comorbidity			0.19			0.76
Hypertension	8(8.6%)	19(12.7%)		8(8.6%)	9(9.7%)	
Diabetes mellitus	3(3.2%)	9(6.0%)		3(3.2%)	3(3.2%)	
coronary heart disease	1(1.1%)	4(2.7%)		1(1.1%)	1(1.1%)	
Other	4(4.3%)	6(4.0%)		3(3.2%)	2(2.2%)	
Maximal lesion size(mm)	10.2 ± 4.3	11.5 ± 3.9	0.21	10.2 ± 4.3	10.9 ± 3.7	0.71
Lesion location			0.34			0.68
Right upper lobe	25(27.2%)	40(26.8%)		25(27.2%)	23(25.0%)	
Right lower lobe	19(20.6%)	36(24.1%)		19(20.6%)	21(22.8%)	
Left upper lobe	26(28.3%)	38(25.5%)		26(28.3%)	25(27.1%)	
Left lower lobe	22(23.9%)	35(23.4%)		22(23.9%)	23(25.0%)	
Type of segmentectomy			0.245			0.73
Simple segmentectomy	30(32.6%)	58(38.9%)		30(32.6%)	32(34.7%)	
Complex segmentectomy	62(67.4%)	91(61.1%)		62(67.4%)	60(65.3%)	

**Fig. 3:**
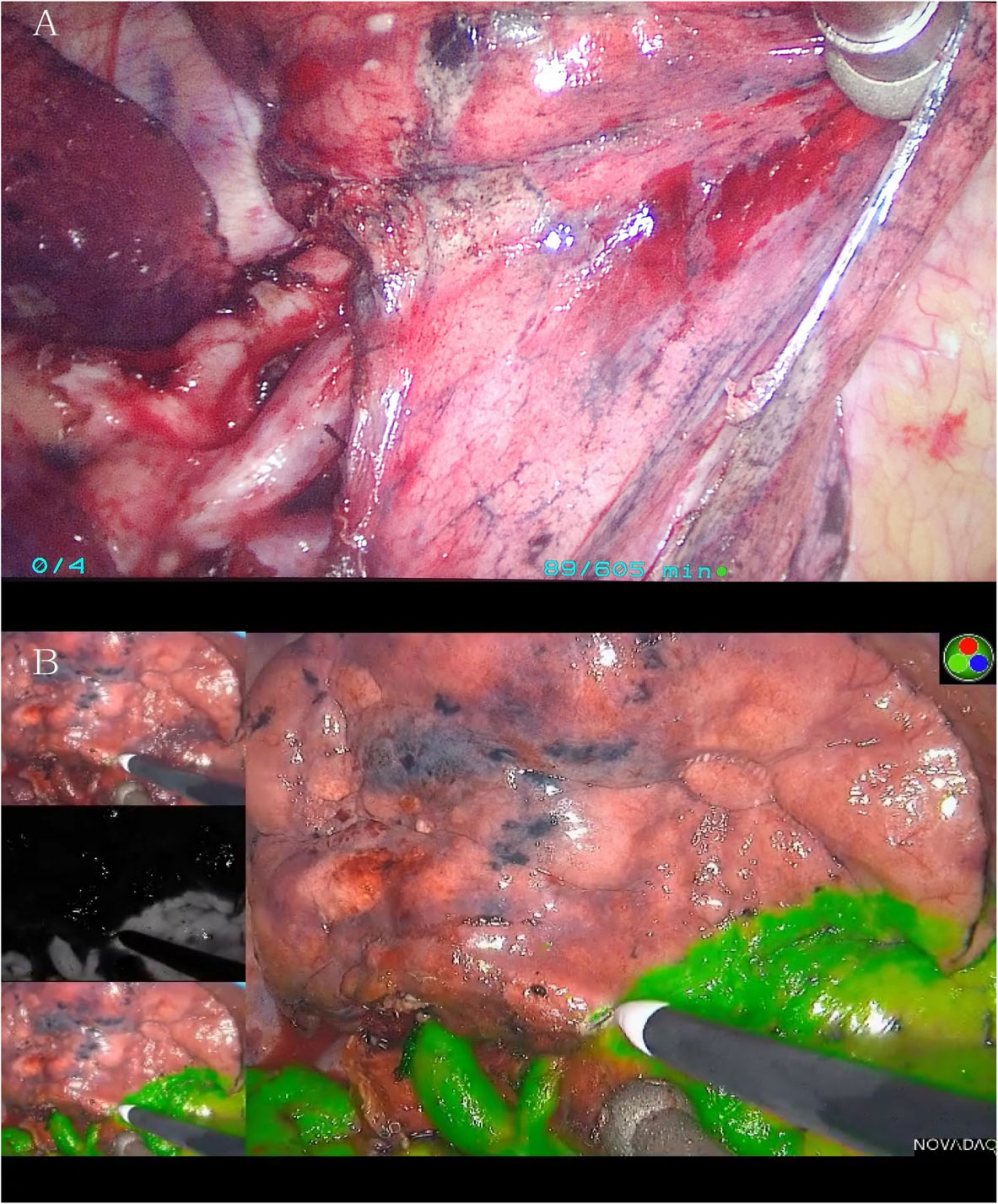
The intersegmental plane visualisation during thoracoscopic segmentectomy. **a**: The intersegmental plane was not clear using inflation-deflation method, **b**: near-infrared intraoperative imaging with indocyanine green is associated with better intersegmental plane visualization

Table 2 illustrated postoperative data. Mean incision size was 3 cm in both group. Mean operative time was 79 min in NIF-VATS group and 96 min in ID-VATS group. The intersegmental plane was not clear in 33 cases of ID-VATS group(Fig. 3a), and no clear boundary was found after prolonged waiting time, including 8 cases of RS^1^ resection, 6 cases of RS^2^ resection, 5 cases of RS^3^ resection, 3 cases of RS6 resection, 1case of LS^1 + 2 + 3^ resection, 4 cases of LS^1 + 2^ resection, 3 cases of LS^3^ resection, 3 cases of LS^6^ resection. Emphysema or pulmonary bullae could be found in Chest CT scan in these patients, these patients were diagnosed as chronic obstructive pulmonary disease. Most cases in NIF-VATS group had better intersegmental plane visualization(Fig. 3b), it was not clear in 8 cases, including 4 cases of RS^1^ resection, 3 cases of RS^2^ resection and 1 case of LS^3^ resection, emphysema could be found in 8 cases.

**Table 2:** Postoperative data of patients who underwent uniportal video-assisted thoracoscopic segmentectomy using NIF imaging or inflation-deflation method.(POD: Post-Operation Day)

Perioperative outcomes	NIF-VATS(*n* = 92)	ID-VATS(*n* = 92)	*P*-value
Operation time (range) (min)	79 ± 29	96 ± 38	**< 0.01**
Blood loss (range) (ml)	46 ± 32	58 ± 36	0.38
Not clear intersegmental plane	8(8.6%)	33(35.8%)	**< 0.01**
Number of lymph node resection	3.2(1–6)	3.4(1–6)	0.85
Drainage duration (range) (days)	4.6 ± 2.9	5.9 ± 3.3	**0.02**
Hospital stays after surgery	5.6 ± 2.1	7.1 ± 3.2	**0.01**
Pathology			0.77
AAH	3	3	
AIS	24	22	
MIA	30	29	
IAC	29	33	
Other benign lesion	6	5	
Postoperative early complications			
Pulmonary infection	1(1.0%)	1(1.0%)	1
Prolonged air leak (> 5 days), n (%)	7(7.6%)	13(14.1%)	**0.01**
Postoperative hemoptysis	21(22.8%)	24(26.0%)	0.25
Irritable cough	19(20.6%)	25(23.5%)	0.35
Atelectasis	0(0.0%)	0(0.0%)	1
Chylothorax	0(0.0%)	0(0.0%)	1
Phrenic nerve palsy	0(0.0%)	0(0.0%)	1
Atrial fibrillation	5(5.4%)	5(5.4%)	1
Pleural effusion required drainage	6(6.5%)	8(8.7%)	0.19
Mortality	0	0	1
Postoperative wound pain			
VAS score(POD1)	2.9(2–6)	3.0(2–7)	0.53
VAS score(POD3)	2.8(1–6)	2.7(1–7)	0.68
VAS score(POD7)	1.6(0–5)	1.7(0–5)	0.47
VAS score(POD30)	1.1(0–3)	1.1(0–3)	0.65
VAS score(POD60)	0.6(0–1)	0.7(0–2)	0.87

Under the guidance of three-dimensional reconstruction and preoperative positioning, the oncological margin length of both groups met the requirements of surgical quality control. There was no case of conversion to thoracotomy in either group.

The intraoperative blood loss, number of lymph node resection showed no statistical difference between the two groups. Histopathologic examination of patients with a pulmonary nodule included AAH, AIS, MIA, IAC and other benign lesion(Table 2).

Postoperative prolonged air leakage (indicating the time of chest tube leakage ≥5 days) was more often observed in ID-VATS group(7.6% in NIF-VATS group and 14.1% in ID-VATS group). Postoperative drainage duration and postoperative hospitalization time was shorter in NIF-VATS group, mean chest tube duration was 4.6 days, mean postoperative hospital stay was 5.6 days. Rate of other complications was not different, including postoperative hemoptysis, atrial fibrillation, pleural effusion, irritable cough(Table 2). There were no serious postoperative complications or death within 30 days after surgery in either group. There was no significant difference of VAS on postoperative day 1, 3, 7, 30, 60.

The median (range) follow-up period was 16(3–31) months for the NIF-VATS group, and 38(8–70) months for the ID-VATS group. All patients were alive. No tumor recurrence or metastasis was noticed during follow-up in both groups.

## Discussion

Segmentectomy is fundamentally a more limited parenchymal resection and is being advocated for early-stage lung cancer. Several clinical studies have shown that the long-term efficacy of segmentectomy for early NSCLC with a diameter of less than 2 cm is similar to that of lobectomy, with more lung function preserved [3]. The advantages of segmentectomy could be preservation of lung function with low frequency of surgical morbidity and mortality, especially for patients with compromised lung function.

Adequate tumor free margin is one of the most important factors in segmentectomy [8, 9]. Precise identification of the intersegmental border is critical for successful anatomical pulmonary segmentectomy. Inflation-deflation method is the widely used, however, it usually takes about 15–20 min to show a intersegmental plane after inflation [7, 10]. For patients with impaired pulmonary function, chronic obstructive pulmonary disease or extensive pleural adhesion, the intersegmental plane was hard to be identified clearly using inflation-deflation method due to decreased lung compliance [11]. In this report, patients with COPD or other factors related with impaired pulmonary function, intersegmental plane was not clear in segmentectomy using inflation-deflation method.

Near-Infrared Fluorescence (NIF) imaging with indocyanine green (ICG) is a recent advancement in minimally invasive segmentectomy [12]. Following ligation of the corresponding segmental artery, vein, and bronchus within the target segment, ICG is then injected into a peripheral vein [13]. The surgical field is visualized with the fluorescence imaging. The target segment, which was now isolated from the pulmonary vasculature and therefore not perfused with ICG, exhibited no fluorescence and remained dark. The remainder of the lung, which was perfused with ICG, became fluorescent green. a clear delineation of the intersegmental plane was then identified as the line separating the dark lung parenchyma from the green lung parenchyma. When pulmonary artery was properly dissected, delineation of the intersegmental plane could be identified about 15 s after injection of ICG. The operation time was significantly shortened. Besides, the brightness of ICG fluorescence mainly depends on both the ICG concentration in blood vessels and the blood flow in the pulmonary artery [14], it is also influenced by the condition of the pulmonary parenchyma, including anthoracosis and emphysema [15]. It may be difficult to clearly visualize the entire intersegmental plane using ICG in patients with severe emphysema, as the blood flow in emphysematous regions is quite low.

ICG method did not change operation method, division sequence of brochus, artery or vein depended on type of segmentectomy, however, division started from central in most of cases. ICG method facilitated identification of intersegmental plane and shortening of operation time. When intersegmental plane was not clear(especially in inflation-deflation group), segmental hilum should be opened wider by electrocautery or other energy divice to identify intersegmental vein [16, 17] with more pulmonary tissue incised, it might lead to more air-leak in patients with chronic lung diseases. For patients with impaired lung function, postoperative air leakage was more often observed in ID-VATS group.

For patients with impaired pulmonary function, chronic obstructive pulmonary disease or extensive pleural adhesion, the intersegmental plane was hard to be identified clearly using inflation-deflation method due to decreased lung compliance. Using NIF technique, the visualization of intersegmental plane mainly depended on blood flow in pulmonary artery, it was showed in patients with chronic lung diseases. However, it is also influenced by the condition of the pulmonary parenchyma, including anthoracosis and emphysema.

Whether the target segmental arteries were cut precisely also influence the identification of the intersegmental plane. Recognition of anatomy based on three-dimensional reconstruction is essential for safe and precise anatomical segmentectomy [18]. It allows the surgeon to be guided in classification of individual anatomical patterns, identification of anomalies, and intraoperative navigation for veins, arteries, and bronchi.

NIF imaging could last for about 30 s in clear condition, it seemed to be a little short, but usually enough to mark the plane using cautery by experienced surgeon.

The median duration of postoperative hospital stay was relatively long, hospitalization in China is relatively long because patients usually do not have to pay high costs for prolonged hospitalization. Patients prefer to stay in the hospital for an additional 1 or 2 days after an operation despite having met the criteria for discharge in our hospital.

There were several limitations to this present study. For retrospective nature of the study, the randomization was absent, and selection bias cannot be eliminated. Although geriatric patients underwent segmentectomy seemed to have better peri-operative result, prospective research was needed to further confirm the conclusion. Small sample size and short follow-up time were also the main limitations, long-term and subjective patient outcomes should be established in future studies to assess both peri-operative outcome and oncologic efficacy.

## Conclusion

Compared with inflation-deflation method, segmentectomy using NIF imaging is feasible for patients with chronic lung diseases with better intersegmental plane, shorter operation time, less complications, it might lead to faster recovery.

## Data Availability

The datasets used and/or analysed during the current study are available from the corresponding author on reasonable request.

## Abbreviations

VATS: Video-Assisted Thoracoscopic Surgery
NIF-VATS: Video-Assisted Thoracoscopic Segmentectomy Using Near-Infrared Fluorescence Imaging
ID-VATS: Video-Assisted Thoracoscopic Segmentectomy Using Inflation-Deflation Method
FEV1: Forced Expiratory Volume in the First Second
COPD: Chronic obstructive pulmonary disease
CPFE: Combined pulmonary fibrosis and emphysema
ILD: Interstitial lung disease
IP: Interstitial pneumonia
IPF: Idiopathic pulmonary fibrosis
ASA: American Society of Anaesthesiologists
VAS: Visual Analogue Scale
CT: Computer Tomography
MIA: Minimal Invasive Adenocarcinoma
IAC: Invasive Adenocarcinoma
AIS: Adenocarcinoma In Situ
AAH: Atypical Adenomatous Hyperplasia
PSM: Propensity Score Match
